# Response of gut microbiome and metabolomic profiles to POLYCAN, a β-glucan derived from *Aureobasidium pullulans* SM-2001 in beagles

**DOI:** 10.1186/s40104-026-01381-3

**Published:** 2026-04-10

**Authors:** Vetriselvi Sampath, Kyejin Lee, Minjeong Kim, Young Suk Kim, Dae Hong Min, Kyudong Han, Sungbo Cho, Dae-Kyung Kang, In Ho Kim

**Affiliations:** 1 Department of Animal Biotechnology, Dankook University, Cheonan, 31116 South Korea; 2 Smart Animal Bio Institute, Dankook University, Cheonan, 31116 South Korea; 3 Department of Microbiology, College of Bio-Convergence, Dankook University, Cheonan, 31116 South Korea; 4 Glucan Co., Ltd., Jinju-Si, 52840 Gyeongsangnam-Do South Korea; 5 Cosmax Pet, 624-33 Gwangjang-Ro, Goesan-gun, Chungcheongbuk-Do South Korea; 6 Center for Bio-Medical Engineering Core Facility, Dankook University, Cheonan, South Korea

**Keywords:** Beagle, Firmicute*s*, Histidine metabolism, IGF-1, Pyrimidine metabolism, Serum calcium

## Abstract

**Background:**

The importance of glucan additives has been widely recognized in farm animals. Yet the precise role of POLYCAN, a β-glucan derived from the black yeast *Aureobasidium pullulans* SM-2001, remains limited in companion animals. Therefore, this study aims to evaluate its effect on performance, nutrient digestibility, hematology, and the gut microbiome and serum metabolites in beagle dogs.

**Methods:**

Eight healthy male beagle dogs (8 months old; 10.70 ± 1.79 kg body weight; 3.00 ± 0.15 body condition score) were enrolled in a 10-week study comprising two phases: Phase 1 (weeks 0–4) and Phase 2 (weeks 6–10), separated by a 2-week washout period. The dogs were divided into two groups and fed a control (CON), basal diet and CON diet supplemented with 1,000 mg/d of POLYCAN. Each of two diets were provided using a cross over design for eight weeks, with four beagles assigned to each treatment. During the washout period, all dogs were fed only the commercial basal diet.

**Results:**

Throughout the experimental period, POLYCAN supplementation did not affect growth performance, nutrient digestibility, or fecal pH in beagles. However, serum calcium, insulin-like growth factor-1 (IGF-1), growth hormone, and immunoglobulin G (IgG) concentrations were significantly higher (*P* < 0.05) in the POLYCAN-supplemented group. Alpha-diversity indices of microbial richness and evenness, as well as beta-diversity based on Bray–Curtis dissimilarity and unweighted UniFrac distances, showed no significant differences between treatment group. At the phylum level, Actinobacteria and Proteobacteria were more abundant in the POLYCAN group, followed by Fusobacteria and Bacteroidota. At family level, Lachnospiraceae*, *Ruminococcaceae*, *Coriobacteriaceae, Lactobacillaceae, Peptostreptococcaceae, and Erysipelotrichaceae exhibited higher relative abundances. Furthermore, the core gut microbiota at genus level was dominated by *Micrococcus* and *Fusobacterium*. Untargeted metabolomic analysis also revealed distinct group separation, identifying key metabolites including lumichrome, D-mannitol, and 2′-deoxycytidine. Pathway enrichment analysis indicated alterations in pyrimidine, histidine, and bile acid metabolism with higher metabolite abundance observed in the POLYCAN-treated group.

**Conclusion:**

Overall, our findings validate that adding 1,000 mg/d POLYCAN to canines’ diet could serve as a functional nutraceutical to enhance their immune and gut health without affecting growth and digestion.

## Background

Dogs are widely recognized as the first animal species to be pet, with archaeological evidence indicating that domestication occurred approximately 15,000 years ago [1]. Since then, dogs have closely evolved with humans and have increasingly been exposed to lifestyle-related risk factors similar to those affecting human populations, including suboptimal feeding practices, nutritionally imbalanced diets, and obesity. Collectively, these factors may predispose dogs to chronic disorders and metabolic syndrome.

The gut microbiome, an intricate community of microorganisms inhabiting the gastrointestinal tracts (GI) of animals, exerts a profound influence on the health and well-being of hosts [2]. Conversely, gut metabolome plays a pivotal role in mediating diet–microbiome–health interactions [3]. Recent studies have demonstrated that gut microbial composition and its metabolites serve as robust biomarkers of GI function and general health in dogs [4]. Accordingly, the maintenance of a balanced and resilient intestinal microbial ecosystem is essential for immune regulation, optimal nutrient utilization, and metabolic stability. Dietary interventions can modulate gut microbial composition and metabolites that influence host health outcomes [5]. Among dietary components, fermentable fibers have attracted considerable attention for their ability to modulate gut microbial communities and promote beneficial bacterial populations.

β-Glucans (β-Glu), linear polysaccharides composed of D-glucose monomers linked by β-1,3 and/or β-1,4 glycosidic bonds, are recognized as functional dietary fibers naturally present in the cell walls of cereals, bacteria, yeast, and fungi [6]. Notably, 1,3/1,6-linked glucans from black yeast-like fungus *Aureobasidium pullulans *(*A. pullulans*) contain amino acids, fibrous polysaccharides, and mono- and di-unsaturated fatty acids, including linoleic and linolenic acids [7] are known for its immunostimulatory properties, including immune cell activation and priming effects on intestinal microbial communities [8]. Previously, Stuyven et al. [9] reported that oral administration of β-1,3/1,6-glucan induced temporal changes in total and antigen-specific immunoglobulin A (IgA) and immunoglobulin M (IgM) concentrations in dogs. Similarly, Rychlik et al. [10] observed significant improvements in histopathological characteristics and immune responses in dogs received dietary β-1,3/1,6-D-Glu. Additionally, Luo et al. [11] demonstrated that the inclusion of 100 mg/kg β-Glu from *Agrobacterium* sp. ZX09 (purity ≥ 90%) improved growth performance and intestinal function in weaning pigs. Furthermore, Marchi et al. [12] reported that inclusion of 0.14% of β-Glu from *Saccharomyces cerevisiae* exhibited the highest relative abundance of phyla Firmicutes and lower Proteobacteria in beagle dogs. Given these promising outcomes, we hypothesize that dietary β-Glu may promote canine performance by influencing their gut microbial communities and metabolic functions. Despite this potential, the physiological effects of β-Glu derived from *A. pullulans* in dogs remain limited to date. Accordingly, this study evaluated the impact of POLYCAN, a β-Glu obtained from *A. pullulan* SM-2001, on growth performance, intestinal microbiota, hematological parameters, and metabolomic profiles in dogs with the goal of providing new insights into how POLYCAN could supports gastrointestinal balance and overall well-being, by contributing to advancements in pet health and nutrition.

## Materials and methods

### Animals

Eight healthy neutered male beagles (*n* = 8, age range = 8 months old; body weight = 10.70 ± 1.79 kg; body condition score = 3.00 ± 0.15) were enrolled in a 10-week study comprising two phases: Phase 1 (weeks 0–4) and Phase 2 (weeks 6–10), separated by a 2-week washout period. Prior to the commencement of the study, all dogs underwent health screening, dewormed internally and externally, and received vaccinations against canine distempers and rabies to ensure baseline health. All beagles were fed twice daily, once in the morning between 08:30 and 09:30, and once in the evening between 16:30 and 17:30, and participated in two daily walking sessions as part of their routine care.

### Facilities

**Fig. 1:**
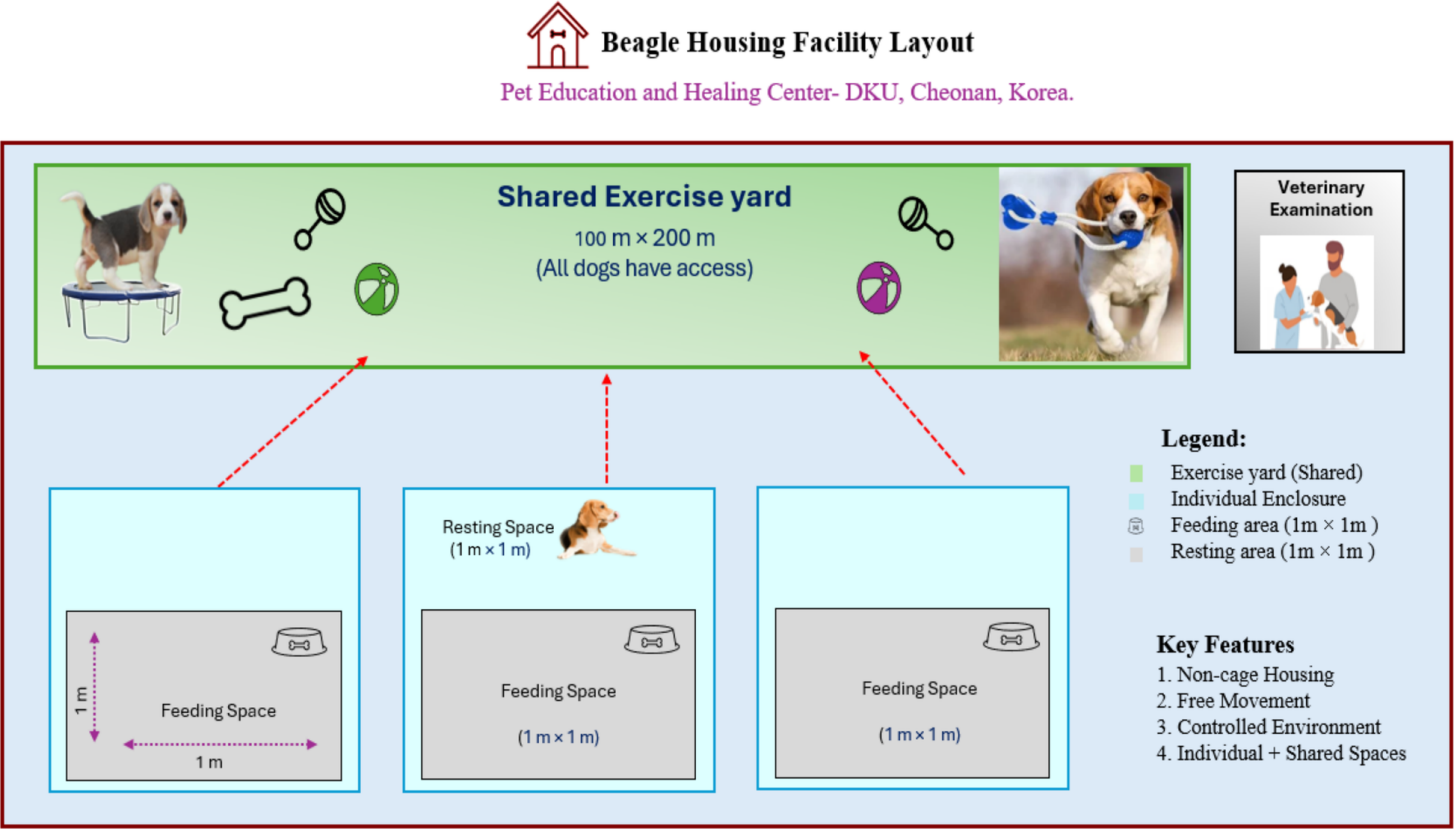
The non-cage housing system allows free movement between individual and shared spaces

This experiment was conducted at the Pet link: Education and Healing Center for Companion Animal Nutrition at Dankook University (Cheonan campus, Republic of Korea). All dogs were housed in a cage-free system that allowed unrestricted movement within designated enclosures (Fig. 1). Each dog had access to an individual feeding area (1 m × 1 m) and a resting space (1 m × 1 m), along with a shared exercise yard measuring 100 m × 200 m. Ambient room temperature was maintained at 23 ± 3 °C with relative humidity of 55% ± 10%, and a 12:12 h light–dark cycle was provided. Enrichment materials such as toys, bedding, and scratching posts were provided to encourage natural behavior and reduce stress. All dogs were supervised by researchers and fresh water was available ad libitum throughout the study.

### Source of β-glucan

The β-1,3/1,6-Glu used in this study, commercially known as “POLYCAN,” was produced via fermentation with *A. pullulans* SM-2001 and supplied by Glucan Co., Ltd. (Jinju-si, Republic of Korea).

### Experimental design, diets and dietary treatments

**Fig. 2:**
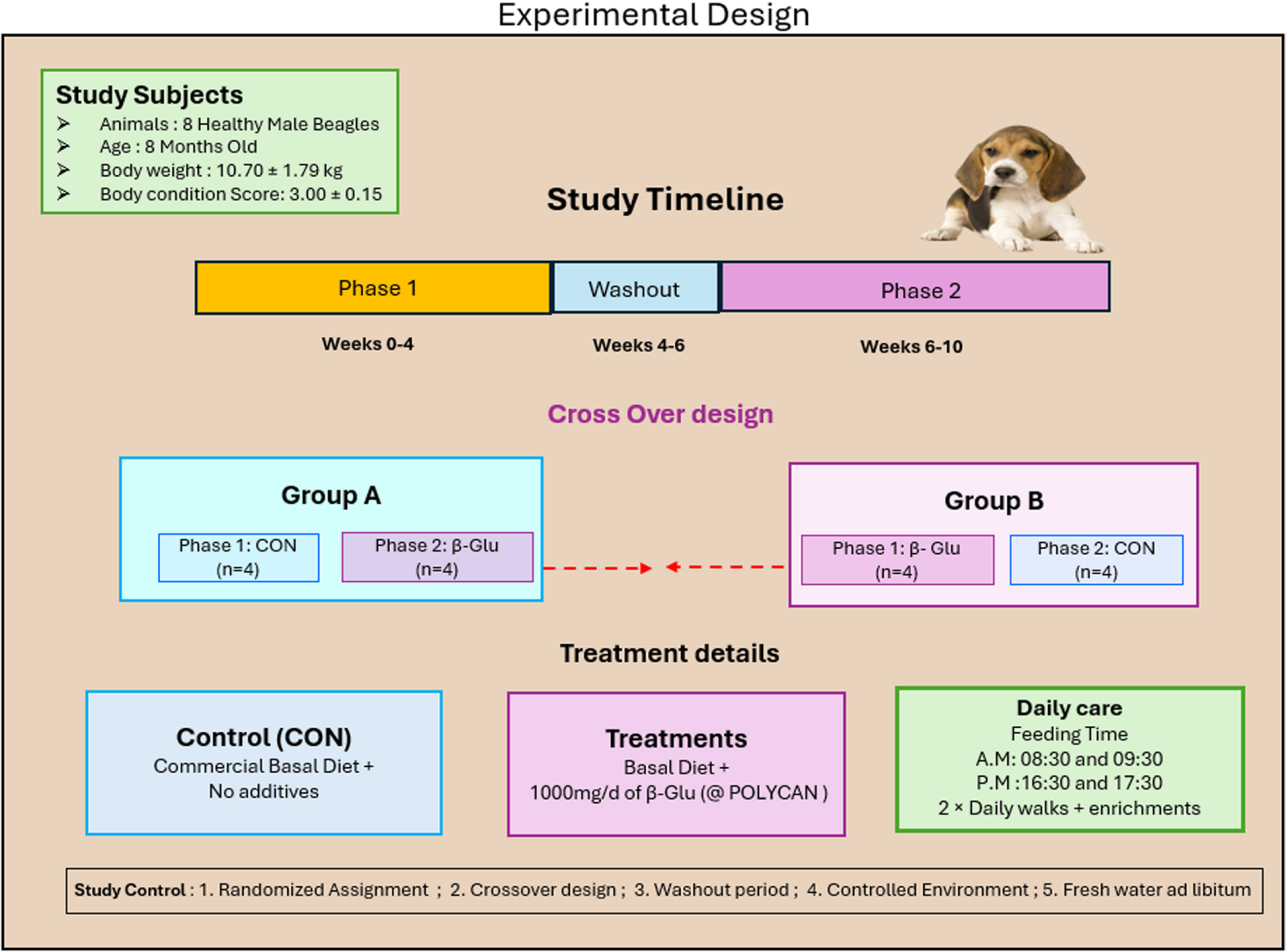
Illustrates the schematic view of the experiment

The study was conducted using a 2 × 2 crossover design over an 8-week feeding period, with four beagle dogs randomly allocated to each dietary treatment per period (Fig. 2). Dogs in the CON group received a commercial basal diet without any additives to serve as baseline, whereas treatment group received the same basal diet top-dressed with POLYCAN at a dose of 1,000 mg/d to assess the comprehensive effects of dietary interventions. A 2-week washout period was included between feeding phases to minimize potential carryover effects and to allow gut microbiota and metabolomic profiles to return towards baseline. During the washout all dogs were fed only commercial basal diet. The ingredients and calculated chemical composition of the basal diets are presented in Table 1.

**Table 1:** Basal diet composition for beagle (fed basis)

Item	Content
Ingredients, %	
Corn	3.00
Wheat	14.82
Rice	8.85
Wheat bran	6.00
Beet pulp	3.63
Soybean meal (45% crude protein)	10.09
Meat bone meal	7.00
Meat meal (60%, low protein)	3.40
Meat meal (70%, high ash)	6.00
Poultry meal	20.00
Tallow	9.20
Poultry fat	5.50
Salt	0.50
Methionine (99%, L-Form)	0.09
Mineral-vitamin premix^1^	1.20
Enzyme	0.03
Dried beer yeast	0.50
Herb	0.12
Yucca	0.02
Antioxidant	0.05
Total	100.00
Calculated value, %	
Dry matter	90.59
Crude protein	32.01
Crude fat	19.97
Crude fiber	2.20
Crude ash	8.79
Calcium	1.96
Total phosphorus	1.26

^1^Formulated to supply a minimum of 0.5 g of magnesium, 1.2 g of sodium, 8.0 g of potassium, 2.3 g of chloride,165 mg of iron, 141 mg of zinc, 7.7 mg of copper, 13 mg of manganese, 0.2 mg of selenium, 1.5 mg of iodine, 0.2 mg of biotin, 1,226 mg of choline, 1.7 mg of folic acid, 45 mg of niacin, 15 mg of pantothenic acid, 7.8 mg of pyridoxine, 6.0 mg of riboflavin, 38 mg of thiamin, and 0.09 mg of vitamin B_12_/kg of diet and to supply 16.4 U of vitamin A, 1.0 U of vitamin D, and 0.18 U of vitamin E/g of diet

### Sampling

Beagles were weighed individually (Electronic scale, model FA2104N, 0.0001 g, Bioprecisa^®^, PR, Brazil) at the start and end of the experiment to determine their initial and final bodyweight (BW). The food intake (FI) was recorded as g of food consumed/kg of BW, to determine average daily feed intake (ADFI) and average daily gain (ADG). Body condition score (BCS) was evaluated using 9-point scale according to the descriptions and illustrations provided by World Small Animal Veterinary Association (WSAVA). The body condition of dogs were classified; “too thin” which is in BCS 1–3 scales had easily visible and palpable bones with no or less fat, and their waist and abdominal tuck was obvious when viewed from top and side, “Ideal” which is in BCS 4 and 5 scales had bones with slight fat covering and an apparent waist and abdomen duck, and “Overweight” and “Obese” which are in BCS 6–9 scales had heavy fat covered ribs, absence of waist and obvious abdominal distension. To evaluate nutrient digestibility, 0.5% chromium (III) oxide as an indigestible marker was top dressed to the diet and fed for a week. Fresh fecal samples were collected from each beagle during the final 5 d of the experiment (i.e., week 10). Samples were obtained within 5 min of defecation, immediately transferred into sterile 10-mL Eppendorf tubes, and properly labeled. The samples were taken to the DKU laboratory within 10 min and subsequently stored at −20 °C for nutrient digestibility analysis and at −80 °C for microbiome analysis. To ensure sample integrity and prevent coprophagy, dogs were confined in pens with one-way slated floors equipped with collection tray. Also, to ensure standardized conditions, 5 mL of fasting blood was collected from each beagle at the end of week 10 via the vena cephalica antebrachii using a 20-gauge needle and the samples were separated into serum and hematology components using serum separator tubes and ethylene diamine tetraacetic acid (EDTA) tubes, respectively. Following centrifugation at 1,811 × *g* for 5–10 min, serum was aliquoted into Eppendorf tubes and stored at −80 °C for further analyses.

### Clinical analysis

#### Nutrient digestibility

The fecal samples were placed in aluminum trays and dried in a forced-convention drying oven (MA035/5, Marconi 1, Brazil) at 60 ºC for 72 h. Later the samples were grounded (Laboratory Sample Mill-SM-450, Harlow, UK), sieved using a 1.0-mm screen, and stored in plastic containers. The nutrient digestibility of dry matter (DM, Method 930.5) and nitrogen (N, Method 954.01) were measured following AOAC [13]. Digestible energy was assessed using bomb calorimetry (Parr 6100; Parr Instrument Co., Moline, IL, USA), while protein content (N × 6.25) was analyzed with a Kjeltec 2300 Analyzer (Foss Tecator™, Hoeganaes, Sweden). Apparent total track digestibility was calculated using the formula: apparent digestibility, % = [(intake of nutrients or energy − fecal output of nutrients or energy)/intake of nutrient or energy] × 100. The fecal moisture content was determined according to Nery et al. [14]. Approximately, 3 g of sample was diluted in 30 mL of distilled water and the fecal pH was measured using a digital benchtop pH meter with an autonomous electrode (Starter 3100, pH Bench, Brazil).

#### Serum profile

Plasma concentrations of calcium (Ca; mg/dL) and phosphorus (P; mg/dL) were determined using colorimetric analysis following complex formation with Arseno-III and via the molybdate reduction method, respectively. Circulating 25-hydroxyvitamin D_3_ [25(OH)D_3_; ng/mL] levels were quantified using a competitive protein binding assay, following the validated canine protocol described by Bardhi et al. [15]. Parathyroid hormone (PTH; pg/mL) was measured using a radioimmunoassay (RIA) specific for intact human PTH (Nichols Institute Diagnostics, USA), exhibiting an inter-assay coefficient of variation of 5.6% at 38 ng/mL and 6.1% at 277 ng/mL, and an intra-assay coefficient of variation of 3.4% at 40 ng/mL and 1.8% at 266 ng/mL. Alkaline phosphatase (ALP; U/L) activity was determined using p-nitrophenyl phosphate as the substrate in a diethanolamine-buffered system. Osteocalcin was measured using Canine OC/BGP (Osteocalcin) ELISA Kit (Code: CNFI00044, Ireland). C-reactive protein (CRP; mg/dL) was measured in lithium heparin plasma using the species-specific Gentian Canine CRP Immunoassay (Gentian AS, Norway) on an ABX Pentra 400 clinical chemistry analyzer (ABX Horiba, France), employing polyclonal chicken-derived antibodies against canine CRP. Plasma insulin-like growth factor-1 (IGF-1; ng/mL) concentrations were determined following acid–ethanol extraction using a radioimmunoassay (RIA) previously validated for dogs [16]. Growth hormone (GH; ng/mL) levels were quantified using a homologous RIA, with intra- and inter-assay coefficients of variation of 3.8% and 7.2%, respectively [17]. Lactate concentrations were determined using the Lactate Pro^®^ LT-1710 analyzer (Arkray Inc., Japan).

Serum biochemical parameters, including creatinine (mg/dL), total protein (g/dL), insulin (µU/mL), albumin (g/dL), and lactate dehydrogenase (LDH; U/L), were measured using a Roche Cobas 6000 c501 analyzer (Roche Diagnostics Co., Ltd., Germany). Serum ferritin (ng/mL) was quantified according to the method described by Andrew et al. [18]. Tumor necrosis factor (TNFα; pg/mL) was measured using dog-specific ELISA plates as per manufacturer’s specifications (Kingfisher Biotech, USA). Superoxide dismutase (SOD; ng/mL) activity was measured using an RX-Daytona automated biochemical analyzer (Randox, Crumlin, UK) with commercial Ransel and Ransod kits (Randox, UK) to assess total antioxidant status. Hematological analyses were performed using a Procyte Dx (IDEXX Laboratories, Maine, USA) automated analyzer to determine the lymphocyte (%), red blood cell (RBC; 10^6^/µL), and white blood cell (WBC; 10^3^/µL). The serum concentrations of immunoglobulin G (IgG; mg/dL) were quantified via enzyme linked immunosorbent assay (ELISA) kit and measured with a microplate reader (Molecular Devices, LLC, CA, USA). The erythrocyte sedimentation rate (ESR; mm/h) was performed using the automated ESR device (MINI-PET, DIESSE, Diagnostica Senese S.p.A.) [19].

#### Fecal microbiome

Approximately 2 g of sample was taken and DNA was extracted with the ReliaPrep gDNA Tissue Miniprep System (PROMEGA Inc., Madison, USA) and subsequently amplified using V3–V4 region-specific primers (341F: 5′-CCTAYGGGRBGCASCAG-3′; 806R: 5′-GGACTACNNGGGTATCTAAT-3′) to produce 16S rRNA gene sequence bacteria under thermal cycling conditions. Next, the amplification products were purified using AMPure XP beads (Beckman Coulter, Switzerland). DNA quality and product size were evaluated on a Bioanalyzer 2100 (Agilent, Palo Alto, CA, USA) using a DNA 7500 chip. The sequencing was performed on the Illumina MiSeq platform with 2 × 300 bp paired end reads (Illumina, Inc., USA) and were processed on the QIIME 2 (Quantitative Insights Into Microbial Ecology 2) platform and data were converted into QIIME 2 artifacts. DADA2 was used for quality filtering, denoising, and removal of low-quality sequences (< Q25), generating amplicon sequence variants (ASVs) via QIIME DADA2-denoise-paired. ASVs were taxonomically classified using a pre-trained naïve Bayes classifier based on the SILVA 138 SSU database.

#### Serum metabolites

For metabolomic profiling, 200 µL of thawed serum was mixed with 800 µL of methanol to precipitate proteins. After vortexing, samples were incubated for 10 min at room temperature and centrifuged at 15,000 × *g* for 15 min at 4 °C. The resulting supernatant was collected and filtered prior to LC–MS analysis. Both hydrophilic and non-polar metabolites were analyzed using hydrophilic interaction liquid chromatography (HILIC) and reverse-phase C18 chromatography, respectively, coupled to a quadrupole time-of-flight (QTOF) mass spectrometer (Agilent Technologies) equipped with a dual AJS electrospray ionization (ESI) source. HILIC separation was performed using an InfinityLab Poroshell 120 HILIC-Z column (2.1 mm × 100 mm, 1.9 µm), while non-polar metabolites were separated using a ZORBAX Eclipse Plus C18 column (2.1 mm × 150 mm, 1.8 µm). For HILIC analysis, the mobile phases consisted of 10 mmol/L ammonium formate in water containing 0.1% formic acid (A) and acetonitrile with 0.1% formic acid (B). A 20-min gradient elution was applied as follows: 0–3 min, 98% B; 3–11 min, 98%–70% B; 11–12 min, 70%–60% B; 12–16 min, 60%–5% B; 16–18 min, 5% B; 18–19 min, 5%–98% B, followed by 1 min re-equilibration. For reverse-phase C18 analysis, the mobile phases were water with 0.1% formic acid (A) and acetonitrile with 0.1% formic acid (B), using a 20-min gradient as follows: 0–2 min, 0% B; 2–10 min, 0–15% B; 10–14 min, 15%–30% B; 14–17 min, 30%–95% B; 17–19 min, 95%–0% B, followed by 1 min re-equilibration. For both chromatographic methods, the flow rate was set at 0.4 mL/min, with an injection volume of 4 µL. Mass spectra were acquired in positive ion mode over an *m/z* range of 50–1,000. A metabolite concentration table containing samples as columns and metabolites as rows was uploaded to the platform. Prior to statistical analysis, serum metabolite data were normalized to reduce technical variation, log-transformed to improve normality, and auto-scaled (mean-centered and divided by the standard deviation). Univariate statistical analysis was performed to identify significantly altered metabolites between experimental groups using Student’s *t*-test, with false discovery rate (FDR) correction applied using the Benjamini–Hochberg. Differential metabolites were visualized using volcano plots, in which the log_2_ fold change was plotted against the −log_10_ (*P* value). Multivariate analysis was conducted using partial least squares–discriminant analysis (PLS-DA), and metabolites contributing most to group separation were identified based on variable importance in projection (VIP) scores greater than 1.0. Metabolite set enrichment analysis was performed using the quantitative enrichment analysis (QEA) module in MetaboAnalyst, which applies the global test algorithm [20]. This method uses a generalized linear model to calculate a Q-statistic that describes the association between metabolite concentration profiles (*X*) and experimental outcomes (*Y*), with the metabolite set Q-statistic calculated as the average of individual metabolite Q-statistics within each set.

### Data analysis

The experimental data were analyzed using SPSS version 22.0 (SPSS Inc., Chicago, IL, USA). Growth performance, nutrient digestibility, fecal pH, and hematological parameters were analyzed using the Linear Mixed Models (LMM) procedure. Dietary treatment and experimental period were used as fixed effects, while dog was included as a random effect to account for the crossover design and repeated measurements within individual animals. The variance–covariance structure was selected based on model fit criteria, and a compound symmetry structure was used. Model assumptions were evaluated by inspection of residual plots and tested for normality. Results are presented as LMM with corresponding standard errors. Statistical significance was declared at *P* < 0.05, and trends at 0.05 ≤ *P* < 0.10. Data of alpha diversity of richness and evenness (Chao1, observed features, Shannon, Simpson, and Pielou’s evenness) were analyzed using the Kruskal–Wallis test (*P* < 0.05). Beta-diversity was analyzed by Principal Coordinate Analysis (PCoA), using the Bray–Curtis dissimilarity method. Differences between groups were analyzed using the PERMANOVA test, considering *P* < 0.05. Serum metabolites analyses were conducted using R version 4.3.2 (2023–10–31) on a Linux operating system.

## Results

The effect of dietary POLYCAN on the growth performance of beagles is presented in Table 2. Dogs fed POLYCAN-supplemented diet showed no significant differences on their growth performance parameters, including BW, ADG, ADFI, or BCS compared with CON. Similarly, there were no (*P* > 0.05) significant differences observed in nutrient digestibility, including DM, N, and energy, nor in fecal moisture content or intestinal pH between the two dietary treatments (Table 3).

**Table 2:** The effect of dietary POLYCAN supplementation on growth performance in beagle dogs^a^

Items	CON	POLYCAN	SEM^b^	*P* value
Body weight, kg				
Initial	11.29	11.05	0.84	0.781
Finish	11.92	11.74	0.74	0.833
Average daily gain, g	23	25	4	0.255
Average daily feed intake, g	220	220	-	
Body condition score				
Initial	3.0	3.0	-	
Finish	3.0	3.0	-	

^a^*CON* Normal commercial diet, *POLYCAN* CON + 1,000 mg/d β-Glu

^b^Standard error of least square means

**Table 3:** The effect of dietary POLYCAN supplementation on nutrient digestibility in beagle dogs^a^

Items, %	CON	POLYCAN	SEM^b^	*P* value
Finish				
Dry matter	84.29	84.54	0.40	0.557
Nitrogen	87.63	88.02	0.31	0.333
Energy	88.63	89.41	0.43	0.154
Fecal moisture	72.40	72.65	2.06	0.926
Intestinal pH	6.10	6.14	0.11	0.760

^a^*CON* Normal commercial diet, *POLYCAN* CON + 1,000 mg/d β-Glu

^b^Standard error of least square means

However, dietary supplementation with POLYCAN significantly affected selected blood biochemical and hormonal parameters in beagle dogs (Table 4). Specifically, calcium concentrations, IGF-1, GH, and IgG were significantly increased (*P* < 0.05) in dogs receiving the POLYCAN-supplemented diet compared with those fed CON. While, no significant differences were observed between treatments for serum phosphorus, 25(OH)D_3_, PTH, ALP, osteocalcin, CRP, lactate, creatinine, insulin, total protein, albumin, ferritin, lactate dehydrogenase, TNF-α, SOD, or hematological indices including WBC, RBC, lymphocyte percentage, and erythrocyte sedimentation rate (*P* > 0.05).

**Table 4:** The effect of dietary POLYCAN supplementation on blood profile in beagle dogs^a^

Items	CON	POLYCAN	SEM^b^	*P* value
Finish				
Calcium, mg/dL	9.09^d^	9.59^c^	0.13	0.017
Phosphorus, mg/dL	5.38	5.50	0.22	0.542
25-OH-D_3_, ng/mL	37.26	37.71	1.04	0.685
PTH, pg/mL	6.20	6.17	0.21	0.855
ALP, U/L	44.9	45.8	2.0	0.703
Osteocalcin, ng/mL	52.61	53.37	2.13	0.674
CRP, mg/dL	0.24	0.20	0.04	0.351
IGF-1, ng/mL	78.05^d^	82.74^c^	0.96	0.002
Growth hormone, ng/mL	0.03^d^	0.05^c^	0.003	0.043
Lactate, mg/dL	13.48	13.36	0.47	0.852
Creatinine, mg/dL	0.78	0.81	0.03	0.373
Insulin, µU/mL	16.28	16.25	0.82	0.974
Total protein, g/dL	7.08	7.25	0.12	0.387
Albumin, g/dL	3.20	3.29	0.13	0.555
Ferritin, ng/mL	171.9	171.5	7.7	0.965
LDH, U/L	129.5	128.4	3.4	0.763
IgG, mg/dL	454.1^d^	479.3^c^	4.6	0.003
TNF-α, pg/mL	24.83	24.37	1.20	0.571
SOD, ng/mL	2.08	2.05	0.07	0.589
WBC, 10^3^/μL	10.32	11.08	0.16	0.126
RBC, 10^6^/μL	6.27	6.30	0.07	0.808
Lymphocyte, %	23.28	24.53	1.34	0.634
ESR, mm/h	1.1	1.3	0.2	0.563

^a^*CON* Normal commercial diet, *POLYCAN* CON + 1,000 mg/d β-Glu

^b^Standard error of least square means

^c,d^Means in the same row with different superscripts differ significantly (*P* < 0.05)

**Fig. 3:**
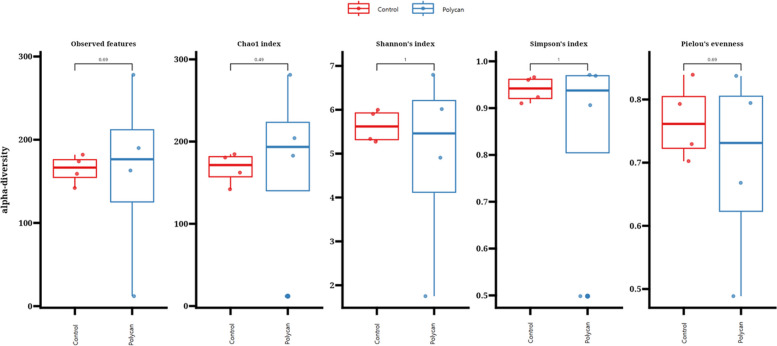
Alpha-diversity indices of the gut microbiota in Control and POLYCAN-supplemented groups. Box plots depict observed features (richness), Chao1 index, Shannon’s diversity index, Simpson’s index, and Pielou’s evenness

**Fig. 4:**
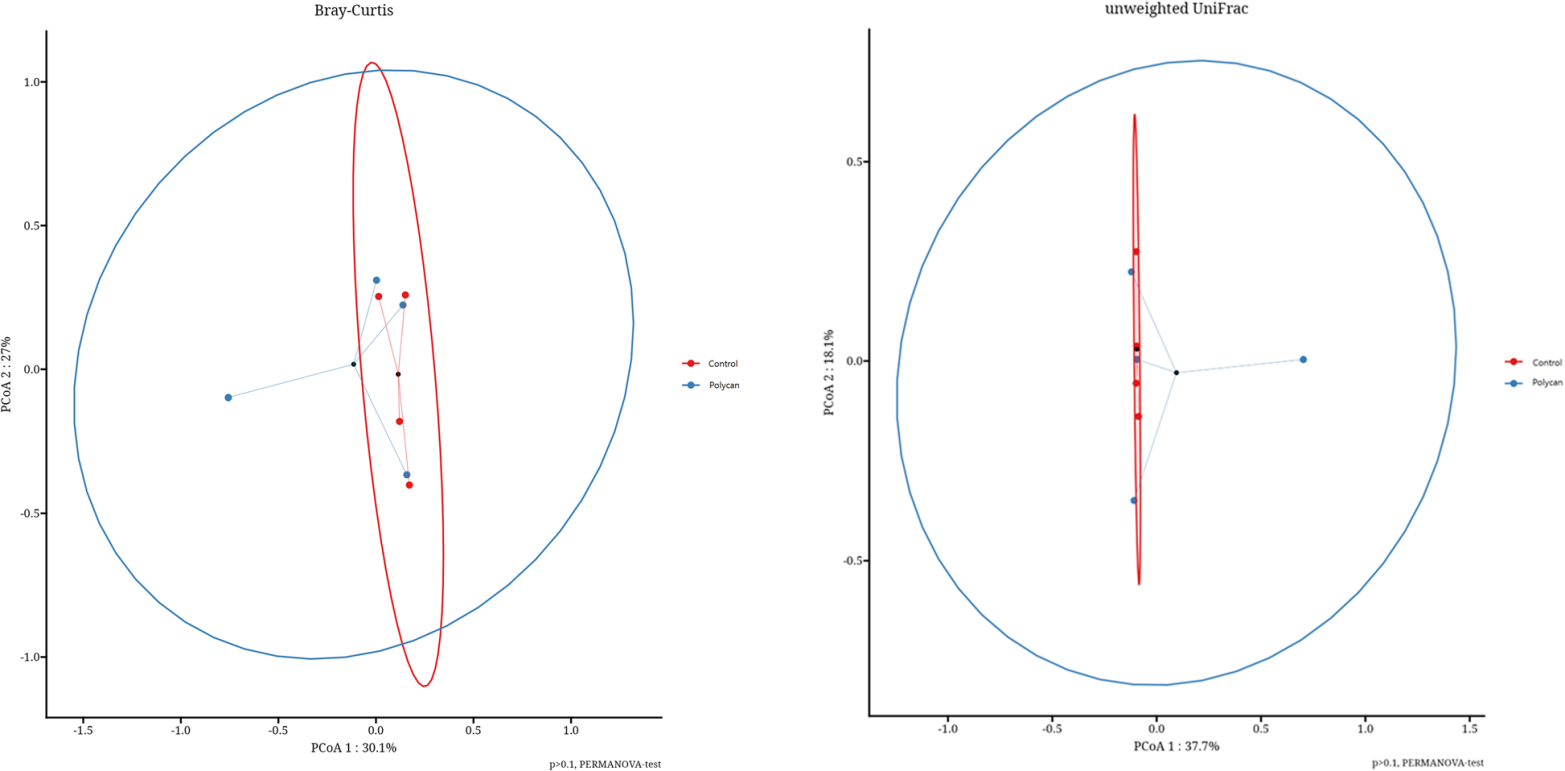
Beta-diversity analysis of gut microbial communities in the Control and POLYCAN-supplemented groups. Principal coordinates analysis (PCoA) plots were generated based on Bray–Curtis’s dissimilarity (left) and unweighted UniFrac distances (right). Each point represents an individual sample, and ellipses indicate the 95% confidence interval for each group. The percentage of variance explained by each principal coordinate is shown on the axes. Statistical differences in community composition between groups were assessed using PERMANOVA, with *P*-values indicated in the plots

**Fig. 5:**
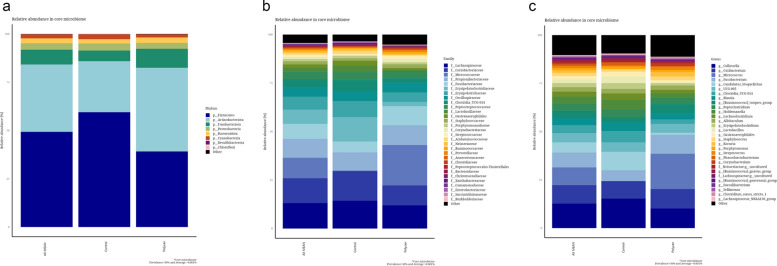
The relative abundance of the core gut microbiome at phylum (**a**), family (**b**) and genus (**c**) level across experimental groups. Stacked bar plots represent the mean relative abundance of dominant bacterial phyla, family, and genera in the ALLMEAN, Control, and POLYCAN-supplemented groups

Also, no significant differences were observed in alpha diversity indices, including observed features, Chao1, Shannon, Simpson, and Pielou’s evenness, between the treatment groups (*P* > 0.05; Fig. 3). Furthermore, PCoA based on Bray Curtis dissimilarity and unweighted UniFrac distances showed no clear separation between the CON and POLYCAN-supplemented groups. Consistent with this, PERMANOVA revealed no significant differences in overall gut microbial community structure between treatments (*P* > 0.05; Fig. 4). Figure 5 illustrates the taxonomic relative abundance of gut microbial communities in beagles at the phylum (a), family (b), and genus (c) levels. At phylum level, the gut microbiome was dominated by Firmicutes and Bacteroidota, followed by Proteobacteria and Actinobacteria, with similar relative abundances observed between the CON and POLYCAN-supplemented groups. Furthermore, the predominant bacterial families across all samples included Lachnospiraceae, Ruminococcaceae, Coriobacteriaceae, Lactobacillaceae, Peptostreptococcaceae, and Erysipelotrichaceae were observed in POLYCAN supplemented group, with no marked alteration in overall family-level microbial composition. At genus level, the core gut microbiota was mainly dominated by *Micrococcus*, *Fusobacterium* followed by *Lactobacillus, Ruminococcaceae* and *Lachnospiraceae*.

**Fig. 6:**
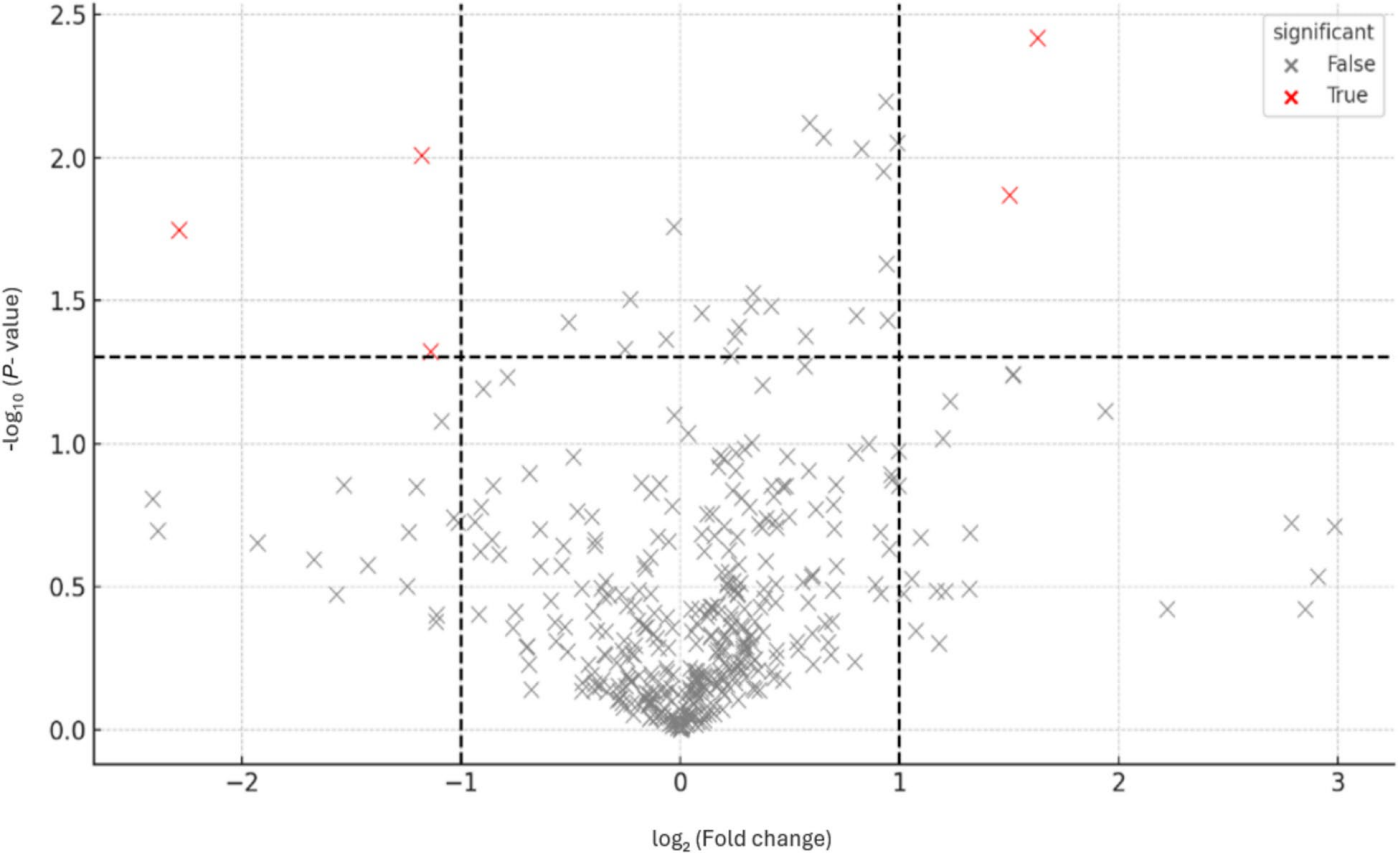
The volcano plot displays differential metabolites between experimental groups based on log_2_ fold change (*x*-axis) and − log_10_(*P* value) (*y*-axis). Each point represents an individual metabolite. Vertical dashed lines indicate the fold-change threshold, and the horizontal dashed line indicates the significance threshold (*P* < 0.05). The red color indicates significant differences in metabolites between the two groups. The black color indicates no significant difference

**Fig. 7:**
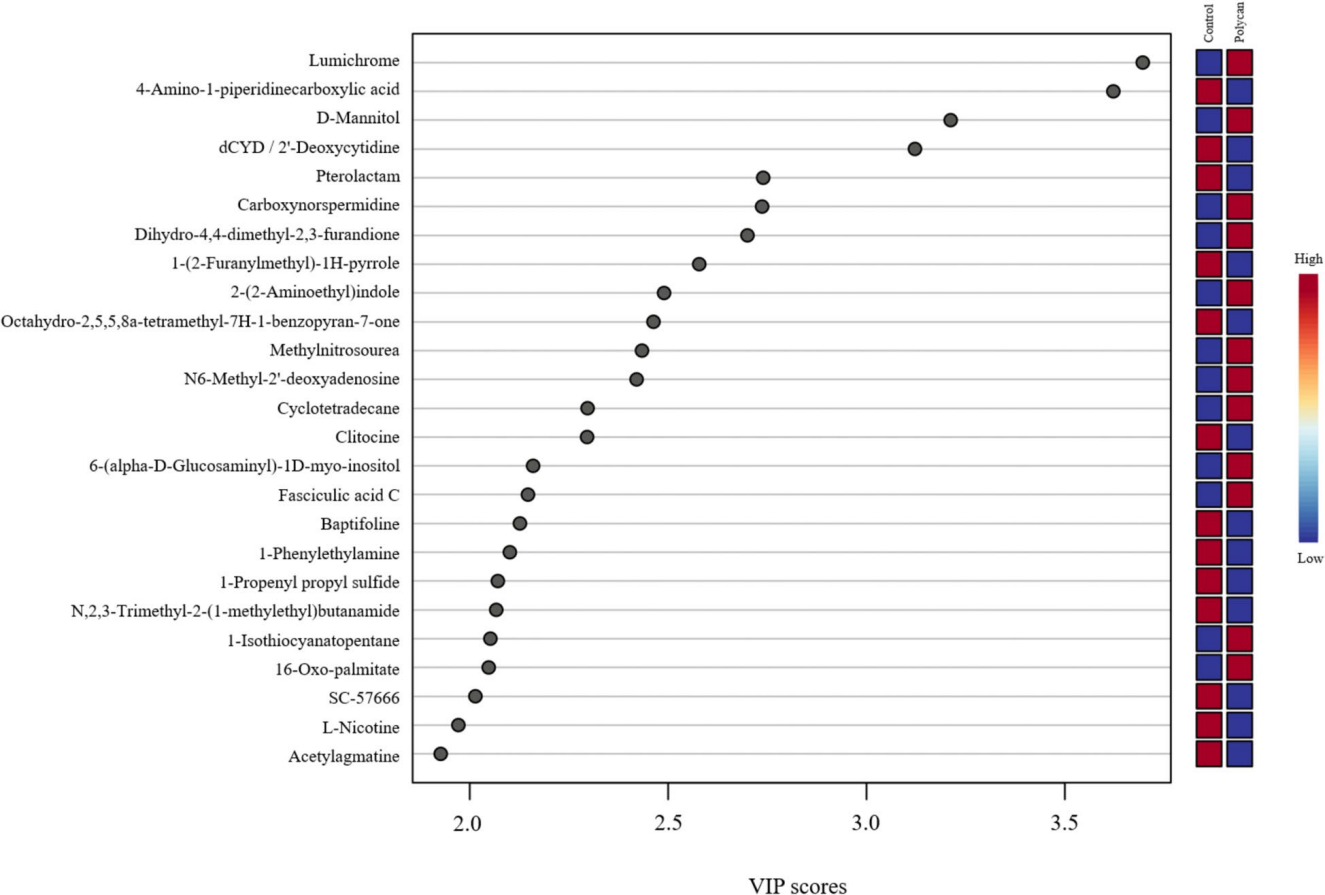
VIP scores of differential metabolites identified by partial least squares–discriminant analysis (PLS-DA). Metabolites with higher VIP values contribute more strongly to discrimination between experimental groups

**Fig. 8:**
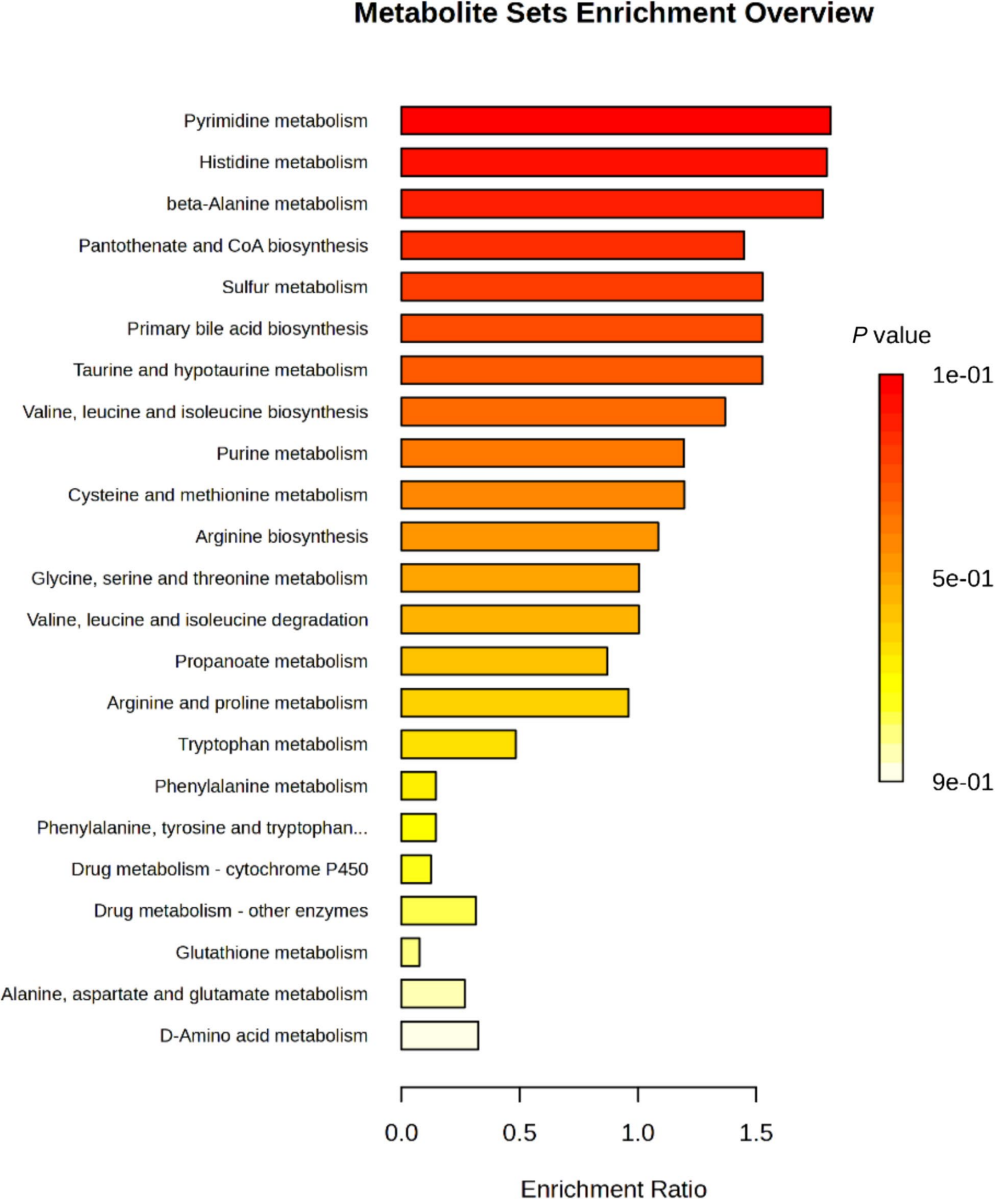
Metabolite set enrichment analysis showing metabolic pathways associated with differential metabolites between experimental groups. Bars represent the enrichment ratio for each pathway, and color intensity corresponds to the *P* value. No pathways remained significant after FDR correction (FDR < 0.05)

Differential metabolite analysis between the CON and POLYCAN groups was visualized using a volcano plot based on log_2_ fold change and −log_10_ (*P*) values (Fig. 6). Using thresholds of [log_2_ fold change] ≥ 1 and *P* < 0.05, a limited number of metabolites were identified as significantly altered between groups. Specifically, several metabolites were significantly upregulated in the POLYCAN group, while a smaller number were significantly downregulated relative to the CON. The majority of detected metabolites clustered near the origin, indicating minimal fold changes. Metabolites with VIP scores greater than 2.0 were considered influential discriminators between groups (Fig. 7). Among these, lumichrome, 4-amino-1-piperidine, D-mannitol, and deoxycytidine/2′-deoxyribose exhibited the highest VIP scores, indicating a strong contribution to group differentiation. Furthermore, quantitative enrichment analysis of the identified metabolites revealed several metabolic pathways with higher enrichment scores, although none reached statistical significance after FDR correction (Fig. 8). Pathways with the highest enrichment statistics included pyrimidine metabolism, histidine metabolism, β-alanine metabolism, and pantothenate and CoA biosynthesis, each showing multiple metabolite hits and elevated Q statistics. The primary bile acid biosynthesis, taurine and hypotaurine metabolism, sulfur metabolism, and valine, leucine, and isoleucine biosynthesis pathways showed notable enrichment. Amino acid–related pathways such as arginine and proline metabolism, arginine biosynthesis, glycine, serine, and threonine metabolism, and cysteine and methionine metabolism were also represented among the enriched pathways.

## Discussion

β-Glu has been used as functional dietary components for enhancing animal health and performance [21]. Yet the inclusion of POLYCAN supplement in beagle diet showed no improvements in their growth parameters and this finding consistent with Ferreira et al. [21] who observed similar outcome in dogs fed oat β-Glu supplement. In agreement with these observations, Rummell et al. [22] also found no differences in feed intake of dogs received a concentrated yeast-supplement. The proposed reason for the absence of measurable effects on performance was attributed either to the intrinsic properties and physical characteristics of the feed ingredients or to differences arising from feed processing techniques [23]. Yeast-derived β-Glu have been shown to enhance the nutrient digestibility of host by modulating their small intestinal function [24]. Nevertheless, in the present study, POLYCAN supplementation did not affect nutrient digestibility of dogs, and this finding aligns with Kilburn-Kappeler et al. [25], who observed similar results in dogs fed β-Glu-supplemented diets. In contrast, Chang et al. [26] observed reduced crude protein and DM digestibility in dogs fed L-β-galactoglucan supplementation. Previously, Dikeman and Fahey [27] stated that β-Glu with high solubility and viscosity increase the water-binding capacity of intestinal contents and alter the physical properties of chyme, thereby impairing nutrient digestion and absorption. From this, we hypothesize that the absence of changes in nutrient digestibility might be due to the lower viscosity, different molecular weight, or inclusion level of POLYCAN, which were likely insufficient to substantially modify digesta characteristics within the gastrointestinal tract.

Fecal quality is a critical indicator of gastrointestinal function and overall digestive health in canine [28]. McRorie and McKeown [29] reported that yeast-derived β-Glu increased the stool moisture, as soluble fibers possess a high water-holding capacity. However, in the present study, fecal moisture content and pH remained consistent across dietary treatments. These results agree with Kilburn-Kappeler et al. [25], who reported no differences in fecal pH in dogs fed diet supplemented with yeast β-Glu, and are also consistent with the findings of Chang et al. [26] and Ferreira et al. [21]. The proposed reason for the absence of changes in fecal consistency and pH might be attributed to the carbohydrate molecules which escape fermentation process and exert an osmotic effect to draw more water into the intestinal lumen and consequently reduce fecal consistency [30] however, to fully understand the specific mechanisms behind these outcomes warrants additional research.

Serum biochemical and immunological indices serve as reliable indicators of host physiological and health status, reflecting both metabolic homeostasis and immune competence [9]. The elevated serum calcium, IGF-1, GH, and IgG concentrations in this study suggest that POLYCAN exerts notable immuno-endocrine modulatory effects in dogs, consistent with the established biological activities of β-Glu [31]. Calcium not only constitutes a structural component of bone but also functions as an essential secondary messenger in immune cell signaling. Intracellular calcium flux is crucial for T-cell activation, cytokine production, and antibody secretion [32]. The increase in systemic calcium observed in this study may indicate that POLYCAN enhances calcium absorption and mobilization and/or indirectly regulates calcium homeostasis through immune activation [33]. Indeed, IGF-1 serves as a key anabolic mediator to govern protein synthesis, cellular proliferation, and tissue repair [34]. Its elevation in the POLYCAN-supplemented group may reflect improved metabolic efficiency and immune cell function, as if IGF-1 promoted lymphocyte proliferation and survival [35]. GH contributes to both growth and immune regulation by stimulating IGF-1 production and directly influencing T- and B-cell differentiation. IgG, the predominant circulating immunoglobulin, plays a central role in humoral immunity by mediating pathogen neutralization and enhancing phagocytosis. Furthermore, observed elevations in IGF-1 and GH suggest that POLYCAN supplementation had enhanced endocrine functions associated with growth and metabolism, while the increase in serum IgG supports its immunostimulatory potential. Previous studies have shown that β-1,3/1,6-Glu supplementation transiently elevates immunoglobulin levels in dogs, indicative of enhanced immune surveillance [31]. From this we proposed that POLYCAN may function as a pathogen-associated molecular pattern (PAMP), interacting with receptors such as complement receptor 3 and dectin-1 to activate innate immune signaling pathways in beagles [36, 37].

β-Glu are recognized as prebiotics, which are known to promote the growth of beneficial microbiota in the gastrointestinal tract [38]. In this study the Alpha diversity of richness and evenness showed no substantial changes between two groups. Though, PCoA plot revealed partial separation between the two groups, with PCoA1 and 2 explaining 30.1% and 23.7% of the total variation, respectively, the PERMANOVA test yielded (*P* = 0.14) no difference. We supposed that the spatial distribution was mainly attributed to the compositional differences influenced by POLYCAN supplementation. With respect to population variations across different taxonomic categories, many changes in the relative abundances of phyla*,* families*,* and genera were detected between treatments. When the composition of the intestinal microbiota was compared at the histological level, the differences in the relative abundances of major taxa were mostly insignificant. At the phylum level, Firmicute*s* was the most dominant taxon with an average of 59.5% in the CON group, followed by *Actinobactertia* with an average of approximately 26.4% [39]. In the POLYCAN group, Actinobacteriota increased to 43.3%, becoming the major dominant taxon, while Firmicutes decreased to 39.1%. Overall, Firmicutes and Actinobacteriota accounted for more than 80%–90% of the intestinal microbiota in both groups, whereas the proportions of other phyla, such as Fusobacteriota*, *Proteobacteria, and Bacteroidota*,* were approximately 2%–10% in each group. In general, phylum Firmicutes is beneficial to intestinal health though they are highly heterogeneous and encompasses several phylogenetic groups [40]. In this study, the Erysipelotrichaceae family primarily contributed to the increased relative abundance of Firmicutes, particularly in the POLYCAN-treated group [29]. In humans, members of the Erysipelotrichaceae family have been associated with various metabolic and inflammatory intestinal disorders [41]. However, in dogs, a lower abundance of these bacteria has been observed in healthy individuals compared to those suffering from diarrhea [42]. Therefore, the reduction in Erysipelotrichaceae populations at the family level suggests its benefit in maintaining intestinal health of canines. Moreover, this family appears to be involved in carbohydrate and fiber digestion, contributing to the production of short chain fatty acids [40]. Overall, the modulation of Firmicutes was linked to a decrease in the Fusobacteriaceae genus *Fusobacterium*. In humans, *Fusobacterium* has been implicated in several intestinal disorders, including inflammatory bowel disease [43]. However, evidence regarding its role in dogs remains inconsistent. Vazquez-Baeza et al. [43] demonstrated that dysbiosis patterns in canine inflammatory bowel disease differ substantially from those observed in humans. In this study, *Fusobacterium* was considered beneficial to the intestinal microbiota of dogs [44], whereas Macedo et al. [45] found that obese dogs exhibited a higher relative abundance of this genus compared to lean dogs. Furthermore, *Fusobacterium* abundance has been reported to be negatively correlated with butyric acid production in the intestine [40]. These conflicting findings highlight the complex and context-dependent role of *Fusobacterium* in canine gut health and underscore the need for further investigation to clarify its biological significance. The phyla Bacteroidetes represented the fifth most abundant group in the fecal microbiota of dogs. Earlier studies reported no significant changes in Bacteroidetes abundance of dogs fed yeast-based products or other prebiotics [46]. The two least abundant phyla, Actinobacteria and Proteobacteria, were also identified in this study, consistent with earlier reports [47]. Notably, a moderate reduction in *Actinobacteria* was observed after POLYCAN supplementation, while *Proteobacteria* abundance, was relatively higher in the CON group and decreased following POLYCAN intake, suggesting a protective modulatory effect of the additive on the intestinal microbiota of beagles [48].

The present metabolomic analysis suggests that dietary POLYCAN supplementation induces selective metabolic adaptations rather than broad disturbances in systemic metabolism. The overall separation between the CON and POLYCAN groups, together with low intra-group variability, indicates a consistent metabolic response to POLYCAN intake. Importantly, these changes appear targeted to specific metabolic networks, supporting the notion that dietary POLYCAN acts as a functional component without eliciting adverse metabolic effects. Metabolites contributing most strongly to group discrimination were primarily associated with nucleotide, amino acid, and carbohydrate metabolism. In particular, alterations within pyrimidine-related pathways suggest a shift in nucleotide turnover rather than excessive catabolism. Although elevated pyrimidine degradation has been associated with intestinal stress, the concurrent reduction in cytosine-related metabolites observed here may reflect a compensatory adjustment that supports intestinal epithelial integrity and limits inflammatory signaling. These findings imply that POLYCAN does not exacerbate mucosal stress and may instead promote metabolic balance within the gut environment. Several metabolites linked to β-alanine metabolism and its associated biosynthetic pathways were also affected by POLYCAN supplementation. β-Alanine serves as a precursor for pantothenate and coenzyme A (CoA) synthesis, which are central to cellular energy metabolism and fatty acid oxidation [49]. The observed shifts in β-alanine related intermediates suggest enhanced metabolic flux through pathways supporting CoA biosynthesis, potentially facilitating energy production and metabolic resilience. Although pantothenic acid levels remained unchanged, enrichment of upstream precursors indicates that POLYCAN may influence pathway efficiency rather than vitamin availability. Metabolites related to histidine and muscle-associated amino acid metabolism further support the absence of tissue damage or excessive protein breakdown. Compounds derived from histidine and dipeptide metabolism are commonly associated with muscle turnover; however, their patterns in this study are more consistent with normal metabolic remodeling rather than pathological change. Together, these findings reinforce the safety profile of POLYCAN with respect to muscle metabolism. Modulation of sulfur-containing amino acid metabolism provides additional insight into the potential systemic benefits of POLYCAN. Methionine cycling plays a critical role in methylation reactions, antioxidant defense, and intestinal barrier maintenance. The apparent reduction in homocysteine accumulation in the POLYCAN group suggests improved efficiency of the methionine metabolic circuit. Whereas changes in bile acid related metabolites indicate that POLYCAN intake may influence bile acid transformation and enterohepatic circulation, likely through interactions with the gut microbiota. The reduction in primary bile acids accompanied by the presence of secondary bile acids in the treatment group suggests enhanced microbial conversion, a process associated with improved cholesterol elimination and lipid homeostasis. Because bile acids are tightly linked to gut microbial activity and host metabolism, these shifts may reflect beneficial host–microbiome interactions promoted by PLOYCAN supplementation.

Taurine metabolism, which intersects sulfur amino acid metabolism and bile acid conjugation, showed modest enrichment in response to POLYCAN. Taurine availability is essential for bile acid conjugation and antioxidant defense, and its metabolic regulation may alter intestinal recycling rather than simple changes in synthesis [50]. Enhanced coordination between methionine metabolism and bile acid conjugation may reduce taurine depletion while supporting hepatic and intestinal function. Overall, the metabolomic adaptations observed in this study indicate that POLYCAN supplementation promotes targeted metabolic modulation involving nucleotide turnover, amino acid cycling, energy metabolism, and bile acid transformation. These coordinated changes support gut health, metabolic efficiency, and host–microbiome homeostasis, while the absence of widespread or pathological alterations further underscores the metabolic safety of POLYCAN in beagles.

## Conclusion

Our study validates the inclusion of 1,000 mg/d POLYCAN in the diet of beagles enhances immune function and favorably modulates gut microbiome composition and serum metabolomic profiles. These findings suggest that it could serve as a promising nutraceutical for promoting overall canine health.

## Data Availability

The datasets generated and/or analyzed during the current study are available from the corresponding author upon reasonable request.

## Abbreviations

25- (OH)-D_3_: 25-Hydroxyvitamin D_3_
ALP: Alkaline phosphate
BCS: Body condition score
BW: Body weight
CRP: C-reactive protein
ESR: Erythrocyte sedimentation rate
FI: Food intake
IGF-1: Insulin like growth factor- 1
IgG: Immunoglobulin G
LDH: Lactate dehydrogenase
N: Nitrogen
PTH: Parathyroid hormone
RBC: Red blood cells
SOD: Superoxide dismutase
WBC: White blood cells
β-Glu: β-glucan
